# PET-CT standardized uptake values are increased in focal nodular marrow hyperplasia: a review of indeterminate marrow lesions at a large teaching hospital

**DOI:** 10.1093/bjrcr/uaag004

**Published:** 2026-02-03

**Authors:** Alexander Pearce, Michael Thomas, Fiona Witham

**Affiliations:** Department of Radiology, Queen Alexandra Hospital, Portsmouth PO6 3LY, United Kingdom; Department of Radiology, Queen Alexandra Hospital, Portsmouth PO6 3LY, United Kingdom; Department of Radiology, Queen Alexandra Hospital, Portsmouth PO6 3LY, United Kingdom

**Keywords:** FNMH, haematopoietic island, marrow hyperplasia, bone metastases, benign bone lesions, PET, MRI

## Abstract

Focal nodular marrow hyperplasia (FNMH) is a benign entity that can create diagnostic uncertainty for the radiologist and, if not recognized, can lead to unnecessary intervention or cause interruption in potential treatment due to the need for follow-up imaging for clarification. Current research suggests lesions with a standardized uptake value over 3.6 on fluorodeoxyglucose positron emission tomography and CT scans should be considered metastatic rather than benign. The aims of this study are to analyze the multimodality characteristics of FNMH, educate the reader, and recommend imaging techniques to improve specificity in the absence of histological analysis. Patients who underwent cross-sectional imaging and nuclear medicine studies between 2018 and 2023 were collected, after concerns were raised as to the nature of indeterminate bone marrow lesions. Data were collected on demographics, malignancy, site of index lesion, imaging characteristics, and follow-up. A total of 9 eligible patients were collected. All patients underwent CT and MRI scanning. 8 of the 9 had Positron emission tomography CT (PETCT), of which 7 had standardized uptake values for PETCT exceeding 3.6 using different radiotracers. All but one of the cases were conservatively managed; the remaining case received radiotherapy to their lesion. FNMH can demonstrate higher PET standardized uptake values (SUV) than previously reported in the literature. This has important implications for treatment as the SUV overlaps with those of metastatic bony lesions. Work is needed to improve the distinction between benign and malignant lesions that lie at this threshold of uptake level.

## Introduction

Haematopoietic marrow hyperplasia, also known as focal nodular marrow hyperplasia (FNMH), is the physiological process of yellow to red marrow reconversion, commonly accelerated by various factors including obesity, heavy smoking, long-distance running, and chronic anaemia. These changes are frequently seen in the appendicular skeleton during magnetic resonance imaging (MRI) studies as symmetrical areas of reduced T1-weighted signal (T1w) with reference to fat marrow, but higher than disc or muscle, and regarded as an entirely benign entity. Haematopoietic marrow hyperplasia is also seen in the axial skeleton, where FNMH may be suspected to be a malignant lesion, such as a metastasis, particularly if there is a history of cancer.^1^ This creates diagnostic uncertainty for the radiologist, for which follow-up imaging may be used for clarification, and biopsies may be performed for histopathological confirmation. As a result, the diagnosis of FNMH is often made months after the preliminary finding, causing interruption in potential treatment or undergoing avoidable radiotherapy in some cases.

With growing imaging demand and access to varied imaging modalities, it is increasingly important that radiologists are educated in the imaging features of FNMH to improve our advice to the clinicians and to improve patient care. Positron emission tomography (PET) is proving important in the staging and management of cancer patients, where the use of standardized uptake value (SUV) thresholds to assess the malignant risk of lesions is helpful in guiding the clinician. Current research suggests lesions with an SUV over 3.6 on FDG PET should be considered metastatic, rather than benign entities, such as FNMH.^2^

The aims of this case series are to analyze the inter-modality imaging characteristics of FNMH lesions, including MRI and PET imaging SUV thresholds, educate the reader in the typical imaging appearances, and recommend imaging techniques to improve the specificity in the absence of histological analysis.

## Subjects and methods

Patients were collected between the period of 2018 and 2023 who underwent cross-sectional imaging and were reviewed by the musculoskeletal radiology team after concerns were raised as to the nature of these lesions. These patients were reviewed by the senior musculoskeletal subspecialty author, their imaging analyzed, and the possibility of an FNMH diagnosis was considered as the primary inclusion criterion. Patients with osseous lesions whose features were deemed not typical for FNMH, or more in keeping with another separate entity, were excluded from the study.

The following imaging characteristics are described in the radiological literature as seen in FNMH lesions and have been used to consider whether lesions of concern may be FNMH:^1^^,^^2^

- Bone marrow lesion identified with the following imaging characteristics:
  - MRI:
    - Rounded bone marrow signal abnormality
    - T1w: Hypointense to fatty bone marrow, but hyper/isointense to skeletal muscle and disc
    - T2w: Hypointense to fatty marrow
    - STIR: Isointense or subtly hyperintense to fatty marrow
    - Dixon: significant drop out (>20% on 1.5 Tesla MRI) suggesting fat content within the lesion
  - Computed tomography (CT):
    - There may be faint medullary sclerosis, some fat content, and the presence of trabeculation, but not typical for haemangioma
  - Nuclear medicine:
    - PET SUV (Standardized uptake value) < 3.6
    - Bone scintigraphy: No uptake
- Suspected bone marrow-centred malignancy on MRI or PET (Positron Emission Tomography) studies which had stable imaging characteristics over a minimum 3-month period.

After preliminary review by the senior musculoskeletal radiologist, this yielded 9 patients who continued to undergo follow-up imaging to further characterize or confirm stability of the index lesion. One patient had received radiotherapy treatment for lesions retrospectively identified as FNMH, highlighting the importance of early detection. Information on patient demographics, history of malignancy, site of index lesion, imaging characteristics, and follow up imaging was collected for analysis.

The imaging protocol techniques performed using either Siemens 1.5 Tesla, GE 1.5 or 3.0 Tesla scanners within this MSK radiology department consist of T1w, T2w, and STIR sagittal sequences and T1w, T2w axial sequences. The signal of the lesion core was compared with both skeletal muscle and intervertebral disc and recorded as hypointense, isointense or hyperintense. Dixon T2 protocol (1.5 Tesla) was also performed in 4 patients during follow-up imaging to confirm the presence of fat within the lesion.

Intravenous Gadolinium was not administered for any of these suspected small malignant bone lesions.

PET imaging varied between fluorodeoxyglucose (FDG), choline, and prostate-specific membrane antigen (PSMA), depending on their primary malignancy. The SUV of the lesion was recorded. Bone scintigraphy studies were reviewed as to whether there was any tracer uptake.

CT studies, when performed, were often the preliminary investigation and therefore analyzed retrospectively. It was noted whether the lesion was visible or demonstrated features, such as faint medullary sclerosis. The core lesion Hounsfield units were measured by histogram analysis to determine whether there was any fat content.

No histological analysis was performed at this radiology institution.

## Results

**Figure 1.**
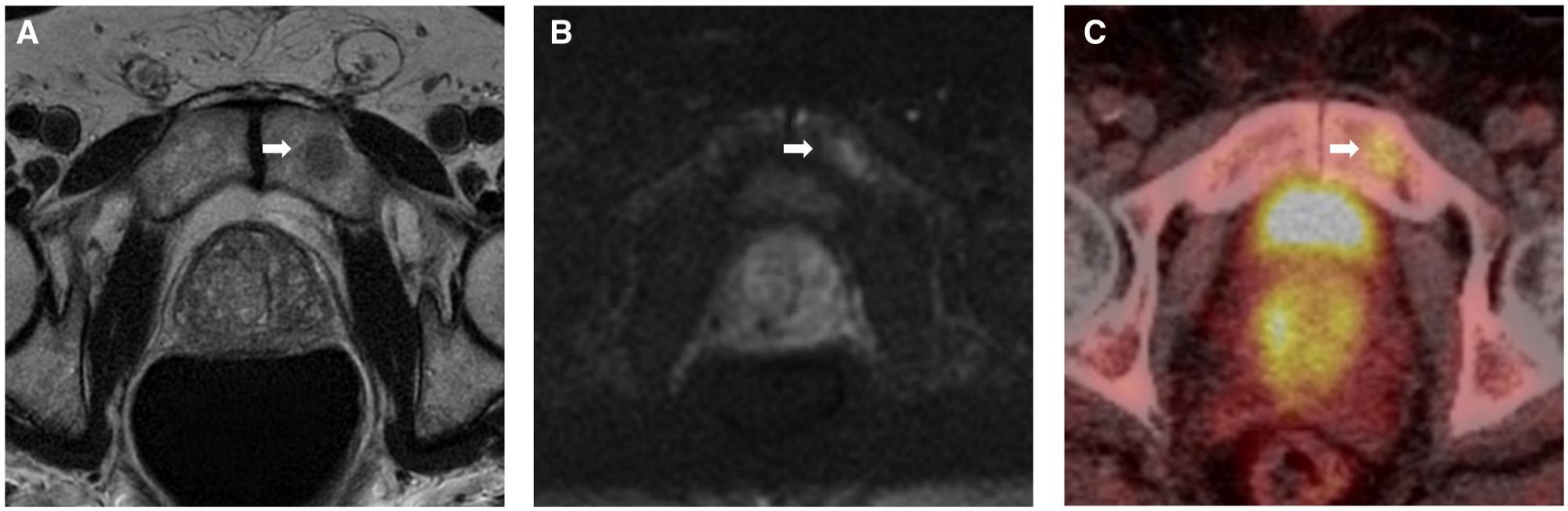
Left pubic body lesion (white arrow) identified within a 75-year-old male undergoing investigation for raised PSA. (A, B) Axial MRI sequences demonstrate intermediate reduced T1w signal (A) and increased B1000 signal (B). (C) Choline PET imaging performed 6 months later identifies an avid lesion with SUV of 5 (C).

**Figure 2.**
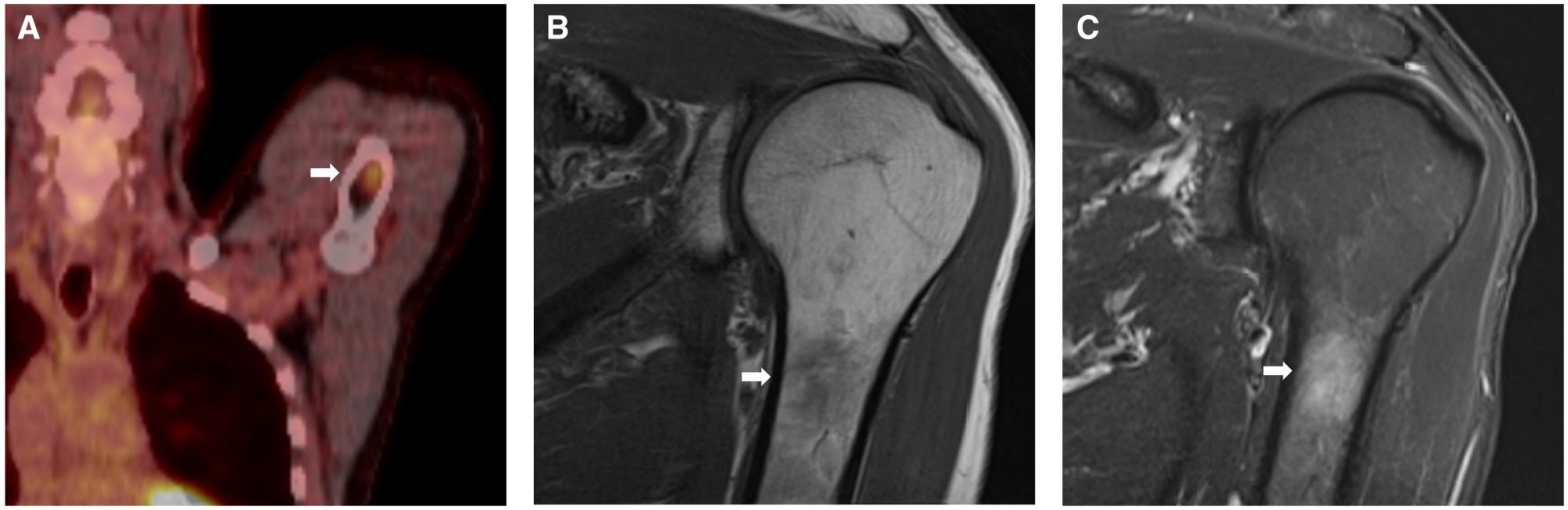
Left proximal humerus lesion (white arrow) in a 70-year-old male with a background of laryngeal malignancy. (A) FDG PET study demonstrated an avid lesion with an SUV of 1.9 (A). (B, C) Sagittal MRI sequences subsequently performed demonstrated reduced T1w signal (B) and increased STIR signal (C) within the lesion.

Nine patients with apparent FNMH at our centre were included in our evaluation (Table 1). The patients were aged between 56 and 77 years at the first visible presentation of the indeterminate marrow lesion. There was a 4:5 female-to-male ratio. Three of the patients had a prior history of known malignancy, and another had a family history of malignancy. The remaining 5 patients had no history of malignancy. The index lesion involved a vertebral body in 7 patients, whilst the remaining 2 patients (Figures 1 and 2) demonstrated involvement of the pubic body and proximal humeral diaphysis respectively.

**Table 1.** The 9 patients with apparent FNMH at our centre included in our evaluation.

Presentation	Index lesion	CT	MRI	Isotope Bone	PET (FDG unless stated)	Subsequent imaging and treatment
**75 M. Presented with raised PSA on a background family history of prostate cancer hence underwent MRI of the prostate.**	Left pubic body	Faintly ill-defined sclerosis	Intermediate reduced T1w and T2w signal. Increased B1000 signal.	No uptake	SUV = 5 (choline)	PSA returned to normal level during initial imaging course. Follow-up MRI 4 years later demonstrated stability. Managed conservatively/not treated.
**70 M. Patient with a history of laryngeal cancer demonstrated a lung lesion on CT. Subsequent PET identified an avid humeral lesion.**	Left humerus	Not seen	Reduced T1w and increased STIR signal.	N/A	SUV = 1.9	Responsible clinician felt the lesion unlikely to be related to the patient’s symptoms. No bone scan performed. Conservatively managed/not treated.
**68 F. Presented with breathlessness. CTPA showed no PE but did show a suspicious lung lesion for which the patient underwent a PET-CT.**	T12 vertebral body	Not seen	Reduced T1w and mildly high STIR signal. Insufficient signal drop-out on Dixon in and out of phase imaging.	N/A	SUV = 5.3	Equivocal radiological appearances on initial MRI. Subsequent CT-guided bone biopsy was unsuccessful. Repeat MRI showed stable lesion characteristics, therefore conservatively managed.
**56 F. History of breast malignancy presented with acute lower back/hip pain. Underwent lumbar MRI.**	L2 vertebral body	Not seen	Reduced T1w/T2w (higher than disc) and subtly increased STIR signal relative to adjacent bone marrow.	No uptake	SUV = 4	Received SABR following MRI and PET imaging. Post-radiotherapy MRI demonstrated signal characteristics consistent with haemangioma or FNMH, rather than metastasis.
**67 F. Investigated with PET for mediastinal lymphadenopathy seen on x-ray, identifying an avid T1 VB lesion.**	T1 vertebral body	Not seen	Reduced T1w/T2w (higher than disc) and subtle increased STIR signal relative to adjacent bone marrow.	N/A	SUV = 4.1	Lesion followed up with x2 stable MRI, the most recent suggesting FMNH. No radiotherapy/treatment. No bone scan.
**75 M. Workup for raised PSA identified an avid lesion on PET.**	L1 vertebral body	Not seen	Subtle reduced T1w, normal T2w and STIR signal. Dixon 40% signal drop-out.	No uptake	SUV = 9.1 (PSMA)	Follow-up PET SUV = 6.3. Vertebral lesion not treated
**77 M. Presents with raised PSA and back pain. Prior prostatectomy and salvage radiotherapy. Underwent PSMA-PET as part of work-up.**	T8 vertebral body	Not seen	Subtle reduced T1w signal when correlating with the PET scan. Dixon 40% signal dropout.	No uptake	SUV = 11.8 (PSMA)	No target for SABR identified at T8 on CT or MRI. Conservatively managed. PSA levels noted to drop down to the normal range during the imaging course.
**76 F. Patient undergoing workup for a solitary lung lesion. Underwent serial PET imaging and MRI, following CT surveillance.**	T9 vertebral body	Not seen	Reduced T1w and T2w, normal STIR signal. Dixon greater than 20% signal drop-out.	N/A	(FDG, SUV 4.6, 2021 and 2023, stable)	No treatment or further surveillance was undertaken with a lesion consistent with FNMH.
**71 M. Presented with severe back pain with high PSA. Underwent MRI spine as part of work-up.**	L2 vertebral body	Faintly ill-defined sclerosis	Focal reduced T1w/T2w signal with more subtle low STIR signal.	N/A	N/A	No bone scans or PET studies undertaken. Conservatively managed/not treated.

All 9 patients underwent CT and MRI scanning of the relevant region, 4 had Dixon sequence MRI including T2 in and out of phase, 8 underwent PET-CT scanning and 4 patients had isotope bone scanning as part of their work-up.

**Figure 3.**
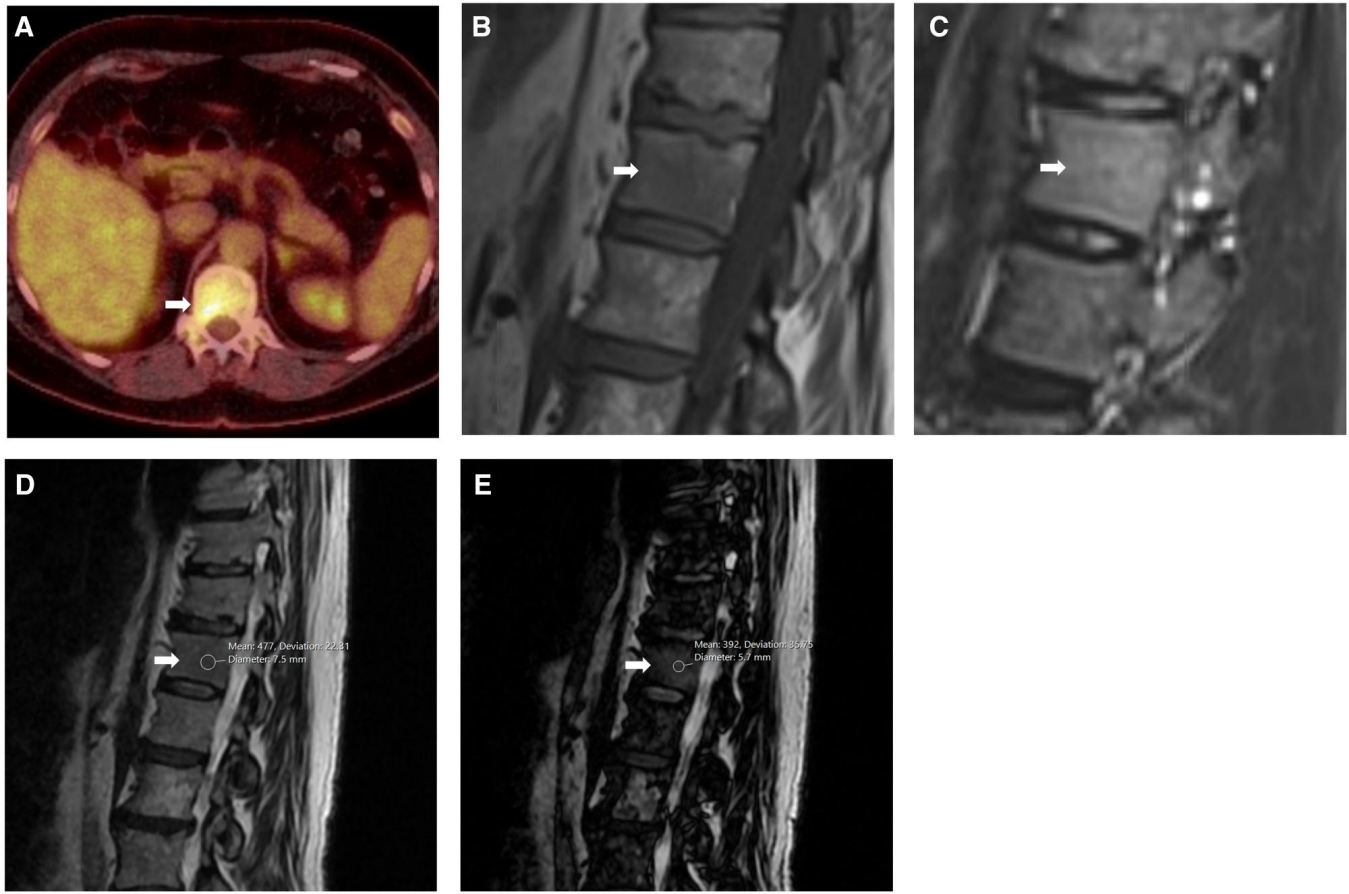
T12 vertebral body lesion (white arrow) in a 68-year-old female undergoing investigation for suspected lung malignancy. (A) Axial FDG PET images demonstrate a focally avid lesion with an SUV of 5.3. (B, C) Sagittal MRI sequences performed 10 days later demonstrates ill-defined reduction in T1w (B) with a subtle increase in STIR (C) signal. (D, E) Sagittal Dixon sequences showed no significant drop-out on in (D) and out (E) of phase imaging. Repeat MRI 6 months later showed stable imaging characteristics.

**Figure 4.**
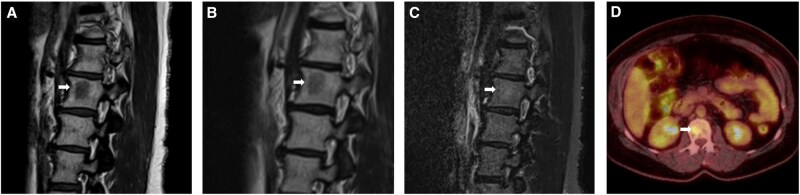
Abnormal L2 vertebral body lesion (white arrow) in a 56-year-old female with a history of breast malignancy. (A-C) sagittal MRI sequences with low T1w signal (A) and low T2w signal (B) lesion, both higher than the adjacent disc signal, and a subtle increase in STIR signal (C). (D) Axial FDG PET study demonstrates an avid lesion with a SUV of 4 within the same period (C).

In 8 out of 9 MRI scans, a definitive focal lesion was seen. Marrow signal variation could only be detected within one patient after correlation with the site of PET uptake. In only 2 of the 9 CT scans was a lesion seen. The SUV values for FDG PET-CT scanning ranged from a low of 1.9 to a high of 5.3, PSMA-PET from 9.1 to 11.8 and a single choline PET-CT scan gave a value of 5. No increased uptake was demonstrated on any of the 4 patients undergoing an isotope bone scan. Seven out of 9 patients had some form of follow-up CT/MRI/PET imaging performed. One patient, in whom the Dixon MRI did not meet the reassuring 20% threshold of signal dropout between in and out of phase, underwent an unsuccessful bone biopsy (Figure 3). In 8 of the cases, the lesions were conservatively managed; however, 1 patient received radiotherapy to their FNMH index lesion (Figure 4).

## Discussion

Imaging follow-up as part of a multi-disciplinary setting between radiologists, orthopaedic surgeons, and oncologists, among other allied health professionals, has been key to distinguishing hyperplastic haematopoietic bone marrow from bony metastatic lesions in both adults and children.^1^ In cases where multimodality imaging with follow-up, including fluid/fat and chemical shift MRI have not yielded conclusive results, percutaneous biopsy for histology of these bone lesions has been confirmatory.^1^^,^^3–6^

Seven out of the total 9 focal nodular marrow hyperplasia cases involved thoracolumbar vertebral bodies, while the remaining lesions were found in the pubic bone and humerus. The anatomical distribution is broadly in line with the majority vertebral body distribution recorded in a series of 53 cases retrospectively reviewed over the course of 13 years.^2^ In addition to the thoracolumbar spine, FNMH lesions have been found in the pelvic bones, and a few have been seen in the cervical vertebrae and long bones.

**Figure 5.**
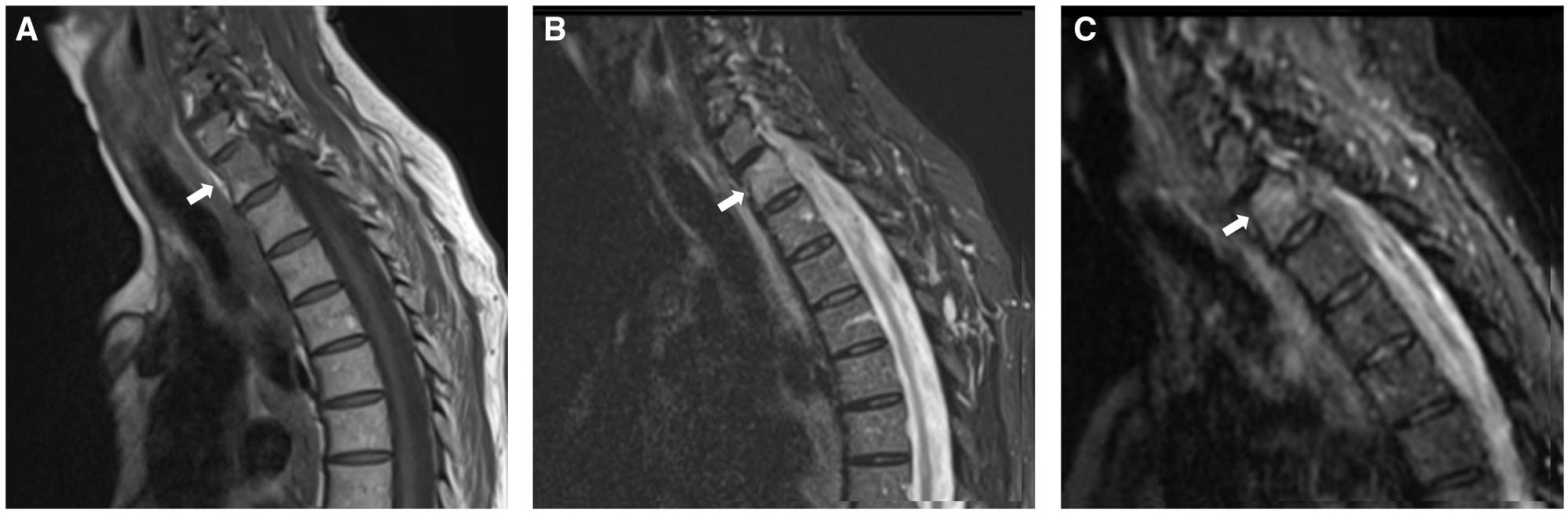
T1 vertebral body abnormal lesion (white arrow) in a 67-year-old female being investigated for mediastinal lymphadenopathy. (A, B) Sagittal MRI sequences demonstrate reduced T1w (A) signal, higher than the adjacent disc signal, and subtle increase in STIR signal (B) within the lesion. (C) Sagittal STIR MRI sequence performed twelve months later demonstrates stable signal characteristics (C).

In our cases, all 9 patients underwent MRI scanning. All lesions demonstrated reduced T1w signal intensity (SI) compared to adjacent marrow but usually higher than that of intervertebral disc and skeletal muscle, such as (Figure 5).

**Figure 6.**
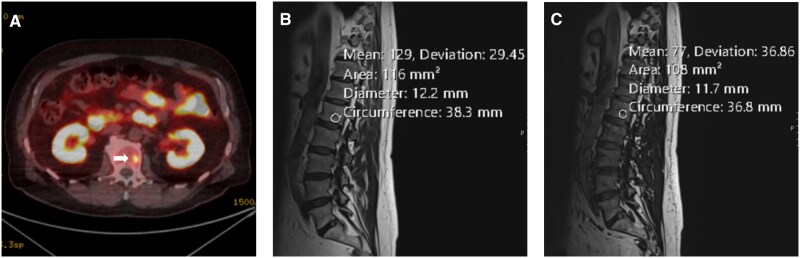
L1 vertebral body lesion (white arrow) within a 75-year-old male undergoing investigations for raised PSA. (A) PMSA PET demonstrates an avid lesion with an SUV of 9.1 (A). (B, C) Sagittal Dixon MRI sequences performed 11 months later demonstrate greater than 40% signal drop-out with mean signal intensities of 129 in-phase (B) vs 77 out-phase (C), suggestive of fat content.

**Figure 7.**
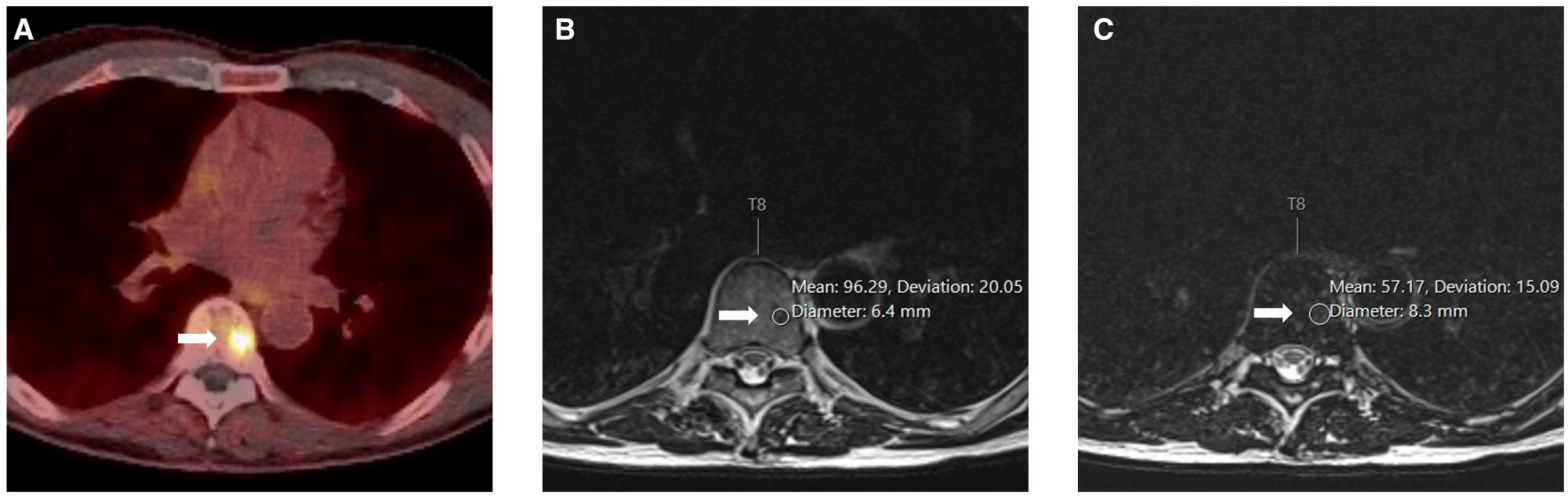
T8 vertebral body lesion (white arrow) in a 77-year-old male undergoing follow-up for previous prostate malignancy. (A) PMSA PET imaging demonstrates the focal avid lesion with SUV of 11.8 in axial formats (A). (B, C) Axial MRI Dixon sequence demonstrates greater than 40% drop-out within the lesion with mean signal intensities of 96.29 in-phase (B) vs 57.17 out-phase (C), suggestive of fat content.

**Figure 8.**
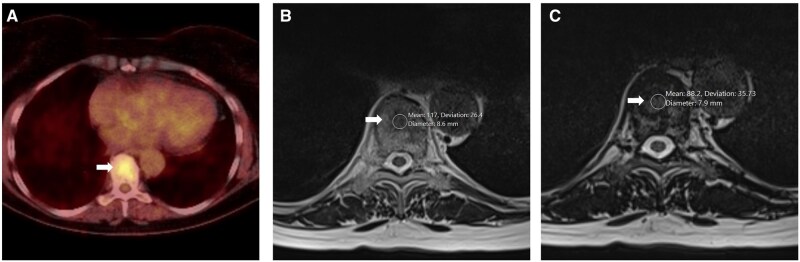
T9 vertebral body lesion (white arrow) in a 76-year-old female undergoing investigation for a solitary lung lesion. (A) Axial FDG PET imaging demonstrates a focal avid lesion with SUV of 4.6, stable on follow up FDG PET 2 years later (A). (B, C) Axial MRI Dixon sequences demonstrated greater than 20% signal drop-out with mean signal intensities of 117 in-phase (B) vs 88.2 out-phase (C), suggestive of fat content.

In 4 cases where standard MRI findings were inconclusive, 3 lesions demonstrated >20% SI dropout between in-phase and out-of-phase Dixon MRI (Figures 6-8), one case being inconclusive on Dixon MRI proceeded to unsuccessful biopsy and then interval MRI, which showed no change (Figure 6).

**Figure 9.**
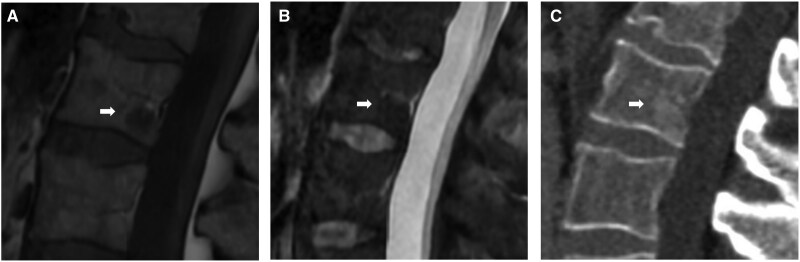
L2 vertebral body lesion (white arrow) in a 71-year-old male undergoing investigation for back pain and raised PSA. (A, B) Sagittal MRI sequences demonstrate focally reduced T1w (A) and STIR (B) signal. (C) Corresponding sagittal CT lumbar spine shows an ill-defined region of subtle sclerosis (C).

Two of the 9 lesions were visible on CT, one faintly hypodense and the other demonstrating faint sclerosis, the latter similar to published cases in the literature (Figure 9). Imaging follow-up was key in most of our cases, as in the majority of previous published case series, including a recent published case series of 53 patients from a UK tertiary orthopaedic centre.^2^ Management of our patients, following seeking of one or more musculoskeletal radiology opinions, leaned predominantly towards follow-up imaging, as opposed to confirmatory biopsy (10%), although an early case was treated with radiotherapy, the lesion having been reported initially as a metastasis. In case series from outside the UK, biopsy was more prevalent.^3^^,^^4^

Focal nodular marrow hyperplasia demonstrating increase uptake on PET studies is previously reported, predominantly on FDG-PET CT studies with a maximum SUV of 3.6.^2^ We have shown that lesions consistent with focal nodular marrow hyperplasia have demonstrated SUVs higher than 3.6, which, up to now, was deemed a threshold for diagnosing metastasis on FDG PET-CTs. In our case, the maximum FDG PET-CT SUV was recorded as 5.3 in a lesion confirmed to be focal nodular marrow hyperplasia on follow-up imaging (following failed bone biopsy).

PET studies utilizing newer tracers such as PSMA, choline and indium chloride, in the context of evaluating indeterminate focal osseous lesions on a background of malignancy, are noted. We have found SUV values associated with the PSMA and choline tracers (SUV maximum 11.8 and 5, respectively), in lesions we believe to be FNMH.

Grunig et al^7^ retrospectively evaluated PSMA-PET scans performed in the context of focal indeterminate bone lesions found in association with prostate cancer. The authors referred to increased unspecific bone uptake (UBU) lesions where there was mild-to-moderate focal uptake of PSMA tracer of an indeterminate lesion (SUV max < 10). Unspecific bone uptake (UBU) lesions were reported in two-thirds of their prostate cancer patients imaged with PSMA-PET. The cases analyzed were mostly in the setting of rib and pelvic lesions (approximately 80%), while vertebral lesions accounted for only 10%, in contrast to our predominantly FDG case series. The focal unspecific uptake regions were called benign in 43% of cases, including marrow hyperplasia, whilst 43% were still considered unclear/indeterminate in nature. The remaining 14% were determined to be malignant (by a mixture of biopsy, biochemical, and imaging follow-up). In our centre, the 2 cases of PSMA-visible lesions were seen in vertebral bodies with maximum uptake SUVs reaching 9.1 and 11.8 were deemed to represent benign focal nodular marrow hyperplasia on follow up imaging (MR with Dixon). It is therefore important to consider SUVs reaching these heights may not reliably predict the likelihood these are malignant bone lesions.

Calabria and their colleagues described the findings associated with choline PET-CT imaging in malignancy. In their study of 169 male patients with prostate cancer, 3.5% of cases were found to have focal bony uptake, with 50% seen in the maxilla. In these cases, the findings were not correlated to bony metastases and were thought to reflect physiological uptake in bone marrow, possibly due to slight enhancement of choline in the reticuloendothelial system.^8^ This has similarity to our case of a patient who underwent choline PET-CT as part of work up for an indeterminate pubic body focal lesion, which demonstrated increased focal uptake with a maximum SUV of 5 (Figure 1). The focal lesion was stable on follow-up MRI 4 years after the initial MRI scan (including Dixon in/out of phase) and thus determined to be of benign character.

Three separate case reports have also described the use of indium chloride scintigraphy in the context of focal nodular marrow hyperplasia.^5^^,^^9^^,^^10^ All patients were men in their 60s diagnosed with oesophageal cancer with bony lesions. Two of these lesions characterized with indium chloride were seen in lumbar vertebral bodies while the remaining lesion was seen in the pelvic iliac bone. In the case report by Okuda et al, a 67-year-old gentleman with oesophageal cancer demonstrated increased FDG PET-CT uptake (maximum SUV 3.3) in the first lumbar vertebral body which was deemed indeterminate between bone marrow hyperplasia and bony metastasis.^9^ The authors of the study described high uptake on indium-chloride scintigraphy as confirming bone marrow reconversion, whilst conventional CT, MRI with chemical shift imaging and FDG PET-CT proved inconclusive. In the absence of any treatment, the raised uptake values in marrow hyperplasia are thought to reflect the metabolic activity associated with reconversion to red marrow.^11^

Our series is limited by the small number (9) of cases in a single centre but illustrates that FNMH can have a PET SUV level (such as 11.8 for PSMA, 5 for Choline, and 5.3 for FDG) that overlaps with those that are widely regarded as suspicious of malignancy. By using other modalities such as MRI, Dixon in/out of phase MRI, CT, isotope bone scanning, and interval MRI, it is usually possible to avoid biopsy of these lesions and to avoid treating such benign lesions as metastatic.

More work is needed to publicize these points and to further explore the overlap between benign and malignant lesion levels of uptake on PET using various tracers.

## Learning points

- Focal nodular marrow hyperplasia is a benign physiological process that demonstrates imaging characteristics similar to malignant lesions, such as metastases. Identifying these lesions early can potentially avoid unnecessary imaging or invasive procedures.
- PET imaging has been previously reported in being able to help distinguish FNMH from malignant lesions by a SUV threshold of 3.6, despite several of our study patients exceeding this value, highlighting the importance of further research in the radiotracers used and the use of other imaging modalities.
- DIXON sequence MRI is a proven useful tool to establish fat within the lesion and therefore benignity, rather than suffering delays with follow-up MRI scans or undergoing a bone biopsy.

## Compliance with ethical standards

Written informed consent was obtained from the patients for publication of this case review, including accompanying images.
